# β-Galactosidase from *Lactobacillus helveticus* DSM 20075: Biochemical Characterization and Recombinant Expression for Applications in Dairy Industry

**DOI:** 10.3390/ijms20040947

**Published:** 2019-02-22

**Authors:** Suwapat Kittibunchakul, Mai-Lan Pham, Anh-Minh Tran, Thu-Ha Nguyen

**Affiliations:** 1 Food Biotechnology Laboratory, BOKU University of Natural Resources and Life Sciences, Muthgasse 18, 1190 Vienna, Austria; suwapatkt@gmail.com (S.K.); mailanpham.22@gmail.com (M.-L.P.); tranminh1703@gmail.com (A.-M.T.); 2 Department of Biology, Faculty of Fundamental Sciences, Ho Chi Minh City University of Medicine and Pharmacy, 217 Hong Bang, Ho Chi Minh City, Vietnam

**Keywords:** *Lactobacillus helveticus*, β-galactosidase, recombinant enzyme, expression systems

## Abstract

β-Galactosidase encoding genes *lacLM* from *Lactobacillus helveticus* DSM 20075 were cloned and successfully overexpressed in *Escherichia coli* and *Lactobacillus plantarum* using different expression systems. The highest recombinant β-galactosidase activity of ∼26 kU per L of medium was obtained when using an expression system based on the T7 RNA polymerase promoter in *E. coli*, which is more than 1000-fold or 28-fold higher than the production of native β-galactosidase from *L. helveticus* DSM 20075 when grown on glucose or lactose, respectively. The overexpression in *L. plantarum* using lactobacillal food-grade gene expression system resulted in ∼2.3 kU per L of medium, which is approximately 10-fold lower compared to the expression in *E. coli*. The recombinant β-galactosidase from *L. helveticus* overexpressed in *E. coli* was purified to apparent homogeneity and subsequently characterized. The *K*_m_ and *v*_max_ values for lactose and *o*-nitrophenyl-β-d-galactopyranoside (*o*NPG) were 15.7 ± 1.3 mM, 11.1 ± 0.2 µmol D-glucose released per min per mg protein, and 1.4 ± 0.3 mM, 476 ± 66 µmol *o*-nitrophenol released per min per mg protein, respectively. The enzyme was inhibited by high concentrations of *o*NPG with *K*_i,s_ = 3.6 ± 0.8 mM. The optimum pH for hydrolysis of both substrates, lactose and *o*NPG, is pH 6.5 and optimum temperatures for these reactions are 60 and 55 °C, respectively. The formation of galacto-oligosaccharides (GOS) in discontinuous mode using both crude recombinant enzyme from *L. plantarum* and purified recombinant enzyme from *E. coli* revealed high transgalactosylation activity of β-galactosidases from *L. helveticus*; hence, this enzyme is an interesting candidate for applications in lactose conversion and GOS formation processes.

## 1. Introduction

Lactic acid bacteria, particularly *Lactobacillus* spp., are well recognized as safe for human consumption, often referred to as probiotics conferring health-promoting properties, and are notable for their potential in food applications [1,2]. *Lactobacillus helveticus*, a homofermentative thermophilic lactic acid bacterium with an optimal growth temperature of 42 °C, is among the most industrially important lactobacilli and plays an important role as a starter culture in the manufacture of fermented dairy products such as cheeses, yoghurts and sour milk [3]. During its growth in milk, *L. helveticus* utilizes lactose as a sole carbon source. The uptake of lactose by this organism occurs through a membrane transport system followed by β-galactosidase-catalyzed intracellular hydrolysis of lactose into the monosaccharides glucose and galactose [4,5,6]. 

Lactic acid bacterial GH2 β-galactosidases are predominantly of the heterodimeric LacLM type, which are encoded by the two overlapping genes, *lacL* and *lacM*, for instance β-galactosidases from *Lactobacillus reuteri* [7], *Lactobacillus acidophilus* [8], *Lactobacillus plantarum* [9], *Lactobacillus pentosus* [10] and *Lactobacillus sakei* [11]. Di- or oligomeric GH2 β-galactosidases of the LacZ type, encoded by the single *lacZ* gene, are sometimes, but not often, found in lactic acid bacteria such as in *Lactobacillus bulgaricus* [12], *Streptococcus thermophilus* [13] and *Streptococcus salivarius* [14]. β-Galactosidases catalyze the hydrolysis and transgalactosylation of β-d-galactopyranosides (such as lactose) [15,16,17]. They catalyze the cleavage of lactose (or related compounds) in their hydrolysis mode, and are thus used in the dairy industry to remove lactose from various products, to prevent crystallization of lactose, to increase solubility of milk-based products, to improve sweetness, to remove lactose from cheese whey and for the production of lactose-free products [13,18,19]. An attractive biocatalytic application is found in the transgalactosylation potential of these enzymes, which is based on their catalytic mechanism, to yield prebiotic galacto-oligosaccharides (GOS) as catalytic products when using lactose as a substrate [15,20]. GOS serve as fermentable substrates for stimulating the growth and activity of probiotics and have been found to modulate the gut microbiota by inhibiting pathogenic bacteria [21,22]. The use of GOS in infant formula and for the development of functional foods is nowadays of significant interest [23]. GOS are also used in a wide range of products such as fermented milk products, breads, jams and beverages [23]. This broad range of food and health-related applications of GOS leads to the challenge in exploring promising β-galactosidases from food-grade sources. 

In response to the profusely use in industrial applications as well as the safety issues concerning enzyme sources, we aimed to overproduce β-galactosidase from *L. helveticus* DSM 20075 (ATCC 15009) in *L. plantarum*, a food-grade host. Most of LacLM-type β-galactosidases are from mesophilic lactobacilli [7,8,9,10,11]; hence, it is of our interest in overproducing a LacLM-type β-galactosidase from a thermophilic source for applications in dairy industry. Our interest is driven by two main reasons: (1) a major drawback of using mesophilic biocatalysts in industrial processes is the threat of microbial contamination and (2) the time needed to obtain high lactose conversion or even complete lactose hydrolysis is reduced at higher process temperatures. In this paper, we describe and compare the heterologous overexpression of *lacLM*-genes encoded β-galactosidase from *L. helveticus* in *Escherichia coli* using an expression system based on the T7 RNA polymerase promoter and in food-grade *L. plantarum* using a lactobacillal expression system. Furthermore, the biochemical properties of the recombinant β-galactosidases pertaining to their ability to produce GOS in biocatalytic processes are reported.

## 2. Results and Discussion

### 2.1. Production of Recombinant β-Galactosidases from L. helveticus in E. coli

**Figure 1:**
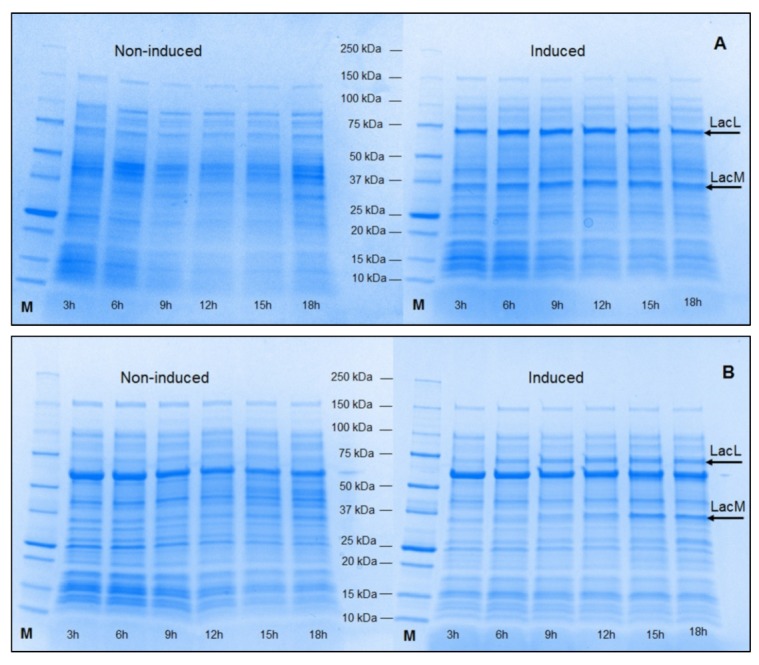

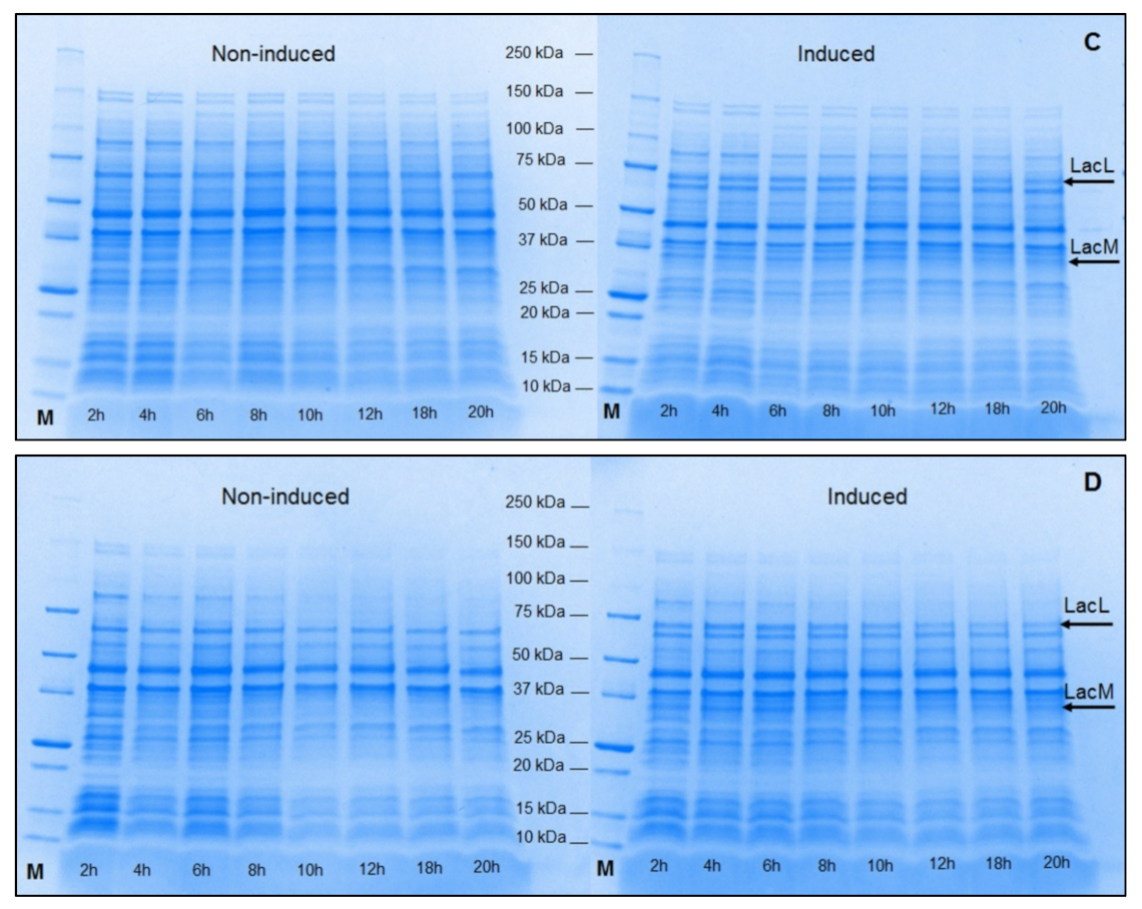
SDS-PAGE analysis of cell-free extract of crude *Escherichia coli* BL21 Star (DE3) (**A**), *E. coli* T7 Express containing the plasmid pGRO7 (**B**), *Lactobacillus plantarum* WCFS1 (**C**) and *L. plantarum* TLG02 (**D**) overexpressing β-galactosidase from *Lactobacillus helveticus* DSM 20075. *E. coli* BL21 Star (DE3) and T7 Express GRO carrying the plasmid pET21lacLMLh were cultivated in 300 mL LB medium; whereas *L. plantarum* WCFS1 and *L. plantarum* TLG02 harboring the plasmids p409lacLMLh and p609lacLMLh, respectively, were cultivated in 500 mL MRS at 30 °C. The cultivation and induction conditions are as described in Materials and Methods and samples were taken at different time points during cultivations (non-induced cultures) and after induction during cultivations (induced cultures). The arrows indicate the LacL and LacM subunits of the recombinant β-galactosidase. M denotes the Precision protein ladder (Biorad, CA, USA).

**Figure 2:**
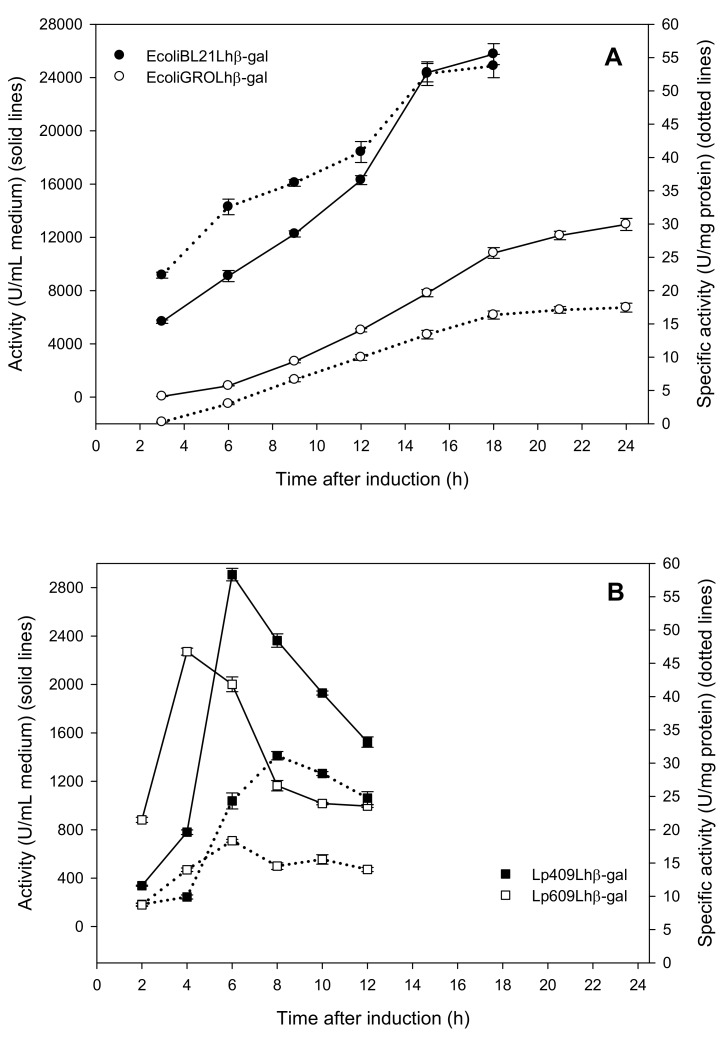
Time course of the cultivations of (**A**) *E. coli* BL21 Star (DE3) and *E. coli* T7 Express containing the plasmid pGRO7 and (**B**) *L. plantarum* WCFS1 and *L. plantarum* TLG02 overexpressing β-galactosidase from *L. helveticus* DSM 20075, resulting in the recombinant β-galactosidases *EcoliBL21Lh*β-gal, *EcoliGROLh*β-gal, *Lp409Lh*β-gal and *Lp609Lh*β-gal, respectively. All data points represent the average value from two independent experiments, and the percentage error was always less than 5%.

The yields of β-galactosidase activity of the wild-type strain of *L. helveticus* DSM 20075 were relatively low. The β-galactosidase yields, as measured in a cell-free extract, obtained after the cultivations of *L. helveticus* DSM 20075 at 37 °C for 24 h, were only ∼ 24.4 ± 0.6 U_*o*NPG_ per L of medium when grown on MRS (*Lactobacillus* broth according to De Man, Rogosa, and Sharpe [24]) containing 2% glucose and ~913.8 ± 11.8 U_*o*NPG_ per L of medium when grown on MRS containing 2% lactose with a specific activity of ~3.0 U/mg (data not shown). Thus, we attempted to overproduce this industrially attractive enzyme using different expression systems. The *lacLM* genes were cloned into the expression vector pET-21d(+). The resulting expression vector, pET21lacLMLh, was then transformed into *E. coli* BL21 Star (DE3) cells and *E. coli* T7 express cells carrying the plasmid pGRO7 containing the chaperones GroEL/GroES. The resulting clones were cultivated in LB medium under inducing conditions (as described in Materials and Methods) to compare the expression yields without and with chaperone co-expression and the SDS-PAGE analyses of cell-free extracts (Figure 1A,B) indicate the production of two recombinant β-galactosidases, *EcoliBL21Lh*β-gal and *EcoliGROLh*β-gal, respectively. The expressed proteins carry a C-terminal His-Tag encoded by the vector. The highest yield were obtained for both systems, without and with the chaperones, when 1.0 mM isopropyl-β-d-thiogalactopyranoside (IPTG) was used for induction and these yields were 25.8 ± 0.8 kU_*o*NPG_ per L of medium with a specific activity of 53.7 ± 1.8 U mg^−1^ for *EcoliBL21Lh*β-gal and 13.0 ± 0.5 kU_*o*NPG_ per L of medium with a specific activity of 17.4 ± 0.7 U mg^−1^ for *EcoliGROLh*β-gal, respectively (Figure 2A). The β-galactosidase activities in non-induced *E. coli* cells were ~350 U_*o*NPG_ per L of medium for *EcoliBL21Lh*β-gal and negligible (0.71 U per L of medium) for *EcoliGROLh*β-gal (data not shown). The expression yield of *EcoliBL21Lh*β-gal is significantly lower to the expression level of LacLM-type β-galactosidase from *L. reuteri* expressed in *E. coli,* which was previously reported to be 110 kU per L of medium [7]; however, it is considerably higher than the yield obtained for β-galactosidase from *L. sakei*, which is also of LacLM-type, expressed in *E. coli* (2.8 kU per L of medium) [11].

Co-expression of the chaperones GroEL/GroES did not show an increase in β-galactosidase activity; in fact, β-galactosidase from *L. helveticus* expressed in *E. coli* cells with chaperones showed a four-fold decrease in activity compared to the activity obtained from the *E. coli* cells without chaperones. We previously reported the expression of two β-galactosidases from *Bifidobacterium breve*, β-gal I and β-gal II, in *E. coli* strains with chaperones, which showed remarkable increase in activity, 30-fold and 14-fold, respectively, compared to the activity obtained from the expression without chaperones [25]. The chaperone family GroEL and its co-chaperone GroES are known to play an essential role in mediating protein folding and preventing protein aggregation [26]. However, it is worth to mention that the β-galactosidases from *Bifidobacterium breve*, β-gal I and β-gal II, are of homodimeric LacZ-type, while β-galactosidase from *L. helveticus* is of heterodimeric LacLM-type; hence, different folding mechanism might explain our observation that the production of β-galactosidase from *L. helveticus* in *E. coli* was not boosted with the co-expression of the chaperones. 

**Figure 3:**
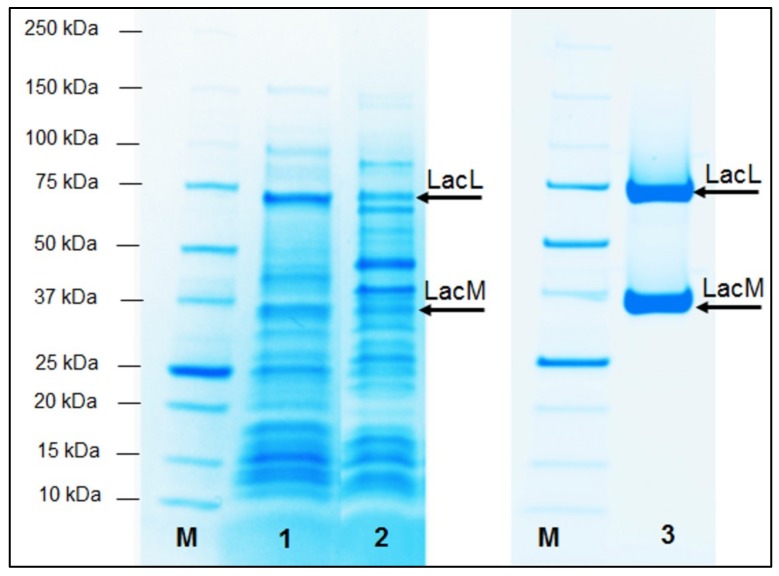
SDS-PAGE analysis of crude and purified β-galactosidase (*lacLM*) from *L. helveticus* DSM 20075 overexpressed in *E. coli* BL21 Star (DE3) and *L. plantarum* WCFS1. Lane 1, whole-cell lysates of *E. coli* BL21 Star (DE3) carrying the plasmid pET21lacLMLh with 1 mM isopropyl-β-d-thiogalactopyranoside (IPTG) induction at 25 °C for 18 h; lane 2, whole-cell lysates of *L. plantarum* WCFS1 containing p409lacLMLh with 25 ng/mL inducing peptide pheromone IP-673 at 30 °C for 8 h; lane 3, purified recombinant β-galactosidase *EcoliBL21Lh*β-gal. The arrows indicate the LacL and LacM subunits of the recombinant β-galactosidase. M denotes the Precision protein ladder (Biorad, CA, USA).

The enzyme *EcoliBL21Lh*β-gal was purified with a single-step purification using a HisTrap HP column and the specific activity of purified enzyme was determined to be ~167.2 ± 6.4 U mg^−1^. The cultivation yielded approximately 25.8 kU of *EcoliBl21Lh*β-gal per L of medium, corresponding to the formation of roughly 155 mg of recombinant *EcoliBl21Lh*β-gal per L of medium and the recombinant β-galactosidase amounted to 32% of the total protein of the host. β-Galactosidase from *L. helveticus* is encoded by *lacLM* genes, which compose of overlapping 1,887 bp (628 amino acid residues) of *lacL* gene and 957 bp (318 amino acid residues) of *lacM* gene with a total length of 2,827 bp (GenBank accession number AJ512877). β-Galactosidase from *L. helveticus* is a heterodimer and the apparent molecular masses corresponding to a large subunit (*lacL*) and a small subunit (*lacM*) were ~75 and ~35 kDa, respectively, as judged by SDS-PAGE (Figure 3) in comparison to reference proteins. These values are in agreement with molecular masses of 73,480.19 and 35,895.49 Da calculated for *lacL* subunit and *lacM* subunit (~109 kDa for *lacLM*), respectively, based on their DNA sequences.

### 2.2. Production of Recombinant β-Galactosidases from L. helveticus in L. plantarum

Lactobacilli have frequently been referred to as “probiotics” because of their health promoting effects to the human gastro-intestinal (GI) tract. Some of the much appreciated effects of lactobacilli have been attributed to their ability to modulate the immune response of the host and to control pathogens by affecting the diversity of the microbial flora in the GI-tract. Several lactobacilli are commensals in the GI tract of humans and animals and are also often used in diverse protocols for fermentation and/or preservation of food and feed [27]. Due to their food-grade status and probiotic characteristics, several lactobacilli are considered as safe and effective cell-factories for food-application purposes [28,29]. *Lactobacillus plantarum* WCFS1 is a well-studied member of the lactobacilli for these purposes. Hence, it was our interest to set out heterologous overexpression of β-galactosidase from *L. helveticus* in this food-grade organism.

The SDS-PAGE analyses of cell-free extracts (Figure 1C,D) confirm the production of two recombinant β-galactosidases, *Lp409Lh*β-gal and *Lp609Lh*β-gal. β-Galactosidase activities of the wild-type host strain *L. plantarum* WCFS1 and the expression strains, *L. plantarum* WCFS1 and *L. plantarum* TGL2, carrying the plasmids p409LacLMLh and p609LacLMLh under non-induced conditions, respectively, were not detected. *L. plantarum* harboring p409lacLMLh, which was constructed based on an erythromycin (*ery*) based pSIP409 expression vector [30,31] (as described in Materials and Methods), showed the highest enzyme yield of ~2.90 ± 0.05 kU_*o*NPG_ of β-galactosidase activity per L of medium and the corresponding specific activity of 24.3 ± 1.2 U mg^−1^ after 6 h of induction (Figure 2B). This yield is somewhat lower when compared with the yields obtained for both LacLM-type and LacZ-type β-galactosidases from lactic acid bacteria. When overexpressing β-galactosidases from *L. reuteri* (LacLM-type), *L. bulgaricus* (LacZ-type) and *Streptococcus thermophilus* (LacZ-type) in the host *L. plantarum* WCFS1 using pSIP409 expression vectors, the yields obtained were ~29 kU, ~22 kU, and ~11 kU of β-galactosidase activity per liter of culture, respectively [12,13,32]. *L. plantarum* harboring p609lacLMLh, which was constructed based on p409lacLMLh and pSIP609, a food-grade alanine racemase (*alr)* based expression vector [32] (as described in Materials and Methods), showed slightly lower expression yield, which was 2.30 kU ± 0.03 kU_*o*NPG_ of β-galactosidase activity per L of medium (Figure 2B), compared to *L. plantarum* harboring p409lacLMLh. This obtained yield is 14-fold lower than the amounts of *L. reuteri* β-galactosidase produced using this *alr*-based system [32]. Lactobacillal pSIP expression systems are strictly regulated and have been proven to overproduce a number of enzymes in *Lactobacillus* hosts [30,33,34]. However, a discrepancy between the yields of expressed proteins was observed as discussed above. Despite higher expression in *E. coli*, the use of the food-grade expression vector (pSIP609) and host organism (*L. plantarum*) has the potential for the production of food-grade enzyme [32,34] and allows the use of crude β-galactosidases in food and feed applications, hence the enzyme costs can be reduced by avoiding laborious and expensive chromatographic steps for the purification of the biocatalyst.

### 2.3. Biochemical Characterization

#### 2.3.1. Enzyme Kinetics

The steady-state kinetic parameters of recombinant β-galactosidase from *L. helveticus* (*EcoliBL21Lh*β-gal) determined for the hydrolysis of *o*-nitrophenyl-β-d-galactopyranoside (*o*NPG) and lactose are presented in Table 1. The *k*_cat_ values were calculated on the basis of the theoretical *v*_max_ values experimentally evaluated by nonlinear regression and using a molecular mass of 109 kDa for the catalytically active product of *lacLM* genes. The experiments with *o*NPG showed substrate inhibition, whereas it was not inhibited by its natural substrate lactose in concentrations of up to 600 mM. Apparently, the hydrolytic potential as well as the affinity of the enzyme for the substrate was found to be far greater with *o*NPG than with lactose. The catalytic efficiency (*k*_cat_/*K_m_*) value for *o*NPG is significantly higher than that of lactose, indicating that *o*NPG is the better substrate for this enzyme. Interestingly, the *K*_m_ value of 15.7 mM determined for lactose is lower compared with the *K*_m_ values reported LacLM-type β-galactosidases from *L. pentosus* (~38 mM) [10] and *L. acidophilus* (~29 mM) [8], but comparable to other LacLM-type β-galactosidases such as *L. reuteri* (~13 mM) [35] and *L. sakei* (~20 mM) [11]. The lower *K*_m,Lac_ value for β-galactosidase from *L. helveticus* suggests the potential use of this enzyme for GOS production and lactose utilization.

#### 2.3.2. Effects of Temperature and pH on Enzyme Activity and Stability

**Figure 4:**
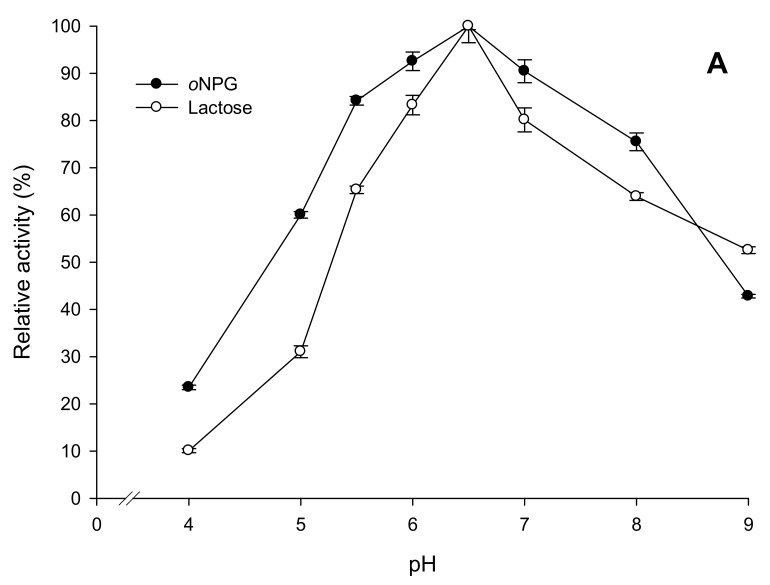

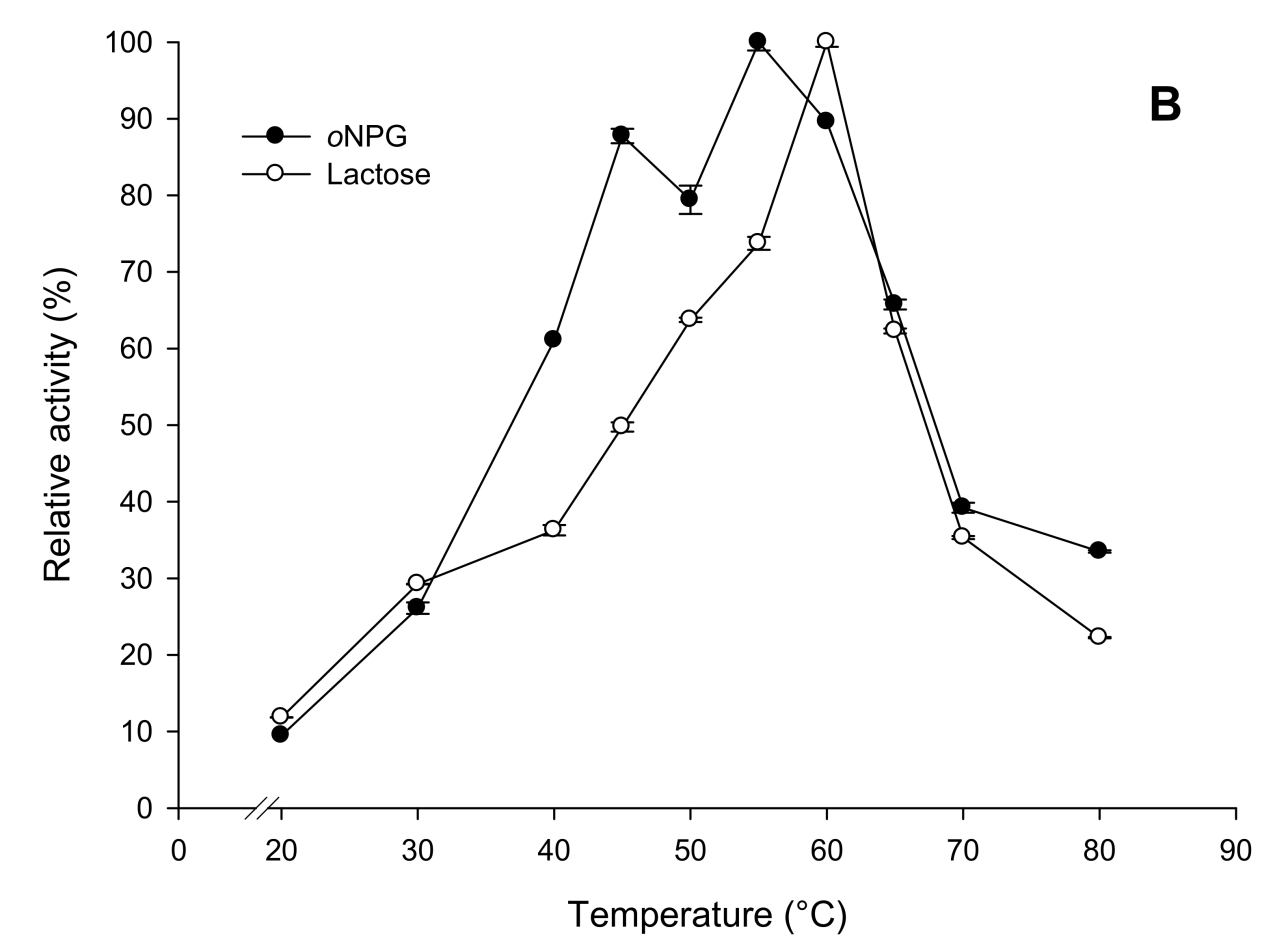
pH optimum (**A**) and temperature optimum (**B**) of recombinant β-galactosidase *EcoliBL21Lh*β-gal using *o*NPG and lactose as the substrates. Relative activities are given in comparison to the maximum activities measured under optimal conditions (100%). All data points represent the average value from two independent experiments, and the percentage error was always less than 5%.

The pH optimum of *EcoliBL21Lh*β-gal is pH 6.5 for both *o*NPG and lactose hydrolysis, and the enzyme exhibits more than 65% of the maximal activity in the pH range of 5.5–8 (Figure 4A). However, the enzyme is most stable at pH 5.5–6.0 with the half-life time (*τ*_1/2_) of activity at pH 5.5 and 6.0 were 816 and 552 h, respectively (Table 2). The pH profile of β-galactosidase from *L. helveticus* showed that it is slightly more active than β-galactosidases from *L. reuteri* [35] and *L. bulgaricus* [12] at mildly acidic pH. The enzyme is very unstable at pH below 5 and above 8 (Table 2).

The temperature optimum of *EcoliBL21Lh*β-gal are 55 °C and 60 °C for *o*NPG and lactose hydrolysis, respectively, under standard assay conditions (pH 6.5 for 10 min) (Figure 4B). The long-term storage of the enzyme could be achieved at 4 °C (*τ*_1/2_ ∼6 months) (Table 2). The enzyme was inactivated very rapidly at temperatures higher than 50 °C as the residual activity after 5 min of incubation at 60, 70, 80 °C was not detected (data not shown). Thermostability of *EcoliBL21Lh*β-gal was significantly improved in the presence of Mg^2+^. Incubation of the enzyme samples in 50 mM NaPB (pH 6.5) with the presence of 1 mM MgCl_2_ at 40 °C and 50 °C showed approximately 15-fold and nine-fold increase in the half-life time (*τ*_1/2_) of activity, respectively, compared to the samples incubated at the same conditions but without MgCl_2_ (Table 2). This positive effect of Mg^2+^ on bacterial β-galactosidases has been demonstrated for both LacLM-type [10,35] and LacZ-type β-galactosidases [12,25,36]. The previous studies on metal-binding sites in the structure of β-galactosidase from *E. coli* has revealed binding sites for Mg^2+^, which are located in the direct vicinity of the active site and might take a part in the catalytic mechanism and stabilization of the enzyme [36,37,38]. Since whey from cheese manufacture contains around 1.5 mM Mg^2+^, the improvement in thermostability of β-galactosidase from *L. helveticus* in the presence of 1 mM MgCl_2_ provides an advantage for the industrial production of GOS using technical substrates such as cheese whey or whey permeates [10]. This helps to enhance the performance of the enzyme without the need of applying exogenous Mg^2+^.

#### 2.3.3. Effects of Various Cations on Enzyme Activity

Various cations were tested with respect to a possible stimulating or inhibitory effect on β-galactosidase activity. These were added in final concentrations of 1–100 mM to the enzyme in Bis-Tris buffer (Table 3). The monovalent cation Na^+^ slightly activated β-galactosidase activity; only approximately 1.5-fold increase in activity was found in the presence of 1 mM and 10 mM Na^+^ compared to the enzyme sample where no cation was added. The presence of 1 mM K^+^ only resulted in a slight activation of the activity, and the enzyme was slightly inhibited in the presence of higher concentrations of K^+^. Interestingly, stimulating and synergistic effect exists in the presence of 1 mM and 10 mM K^+^ together with 1 mM Na^+^, as a two-fold increase in activity was found. The divalent cations Mn^2+^, Mg^2+^, Ca^2+^, Zn^2+^ and Cu^2+^ showed inhibitory effects. It might be necessary to reduce the level of free Ca^2+^ in solution when using lactose-rich substrate such as liquid whey in applications in the dairy industry. However, the application of this enzyme in fluid milk for the production of low-lactose and lactose-free milk should not be an issue because Ca^2+^ is bound to casein.

### 2.4. GOS Formation

**Figure 5:**
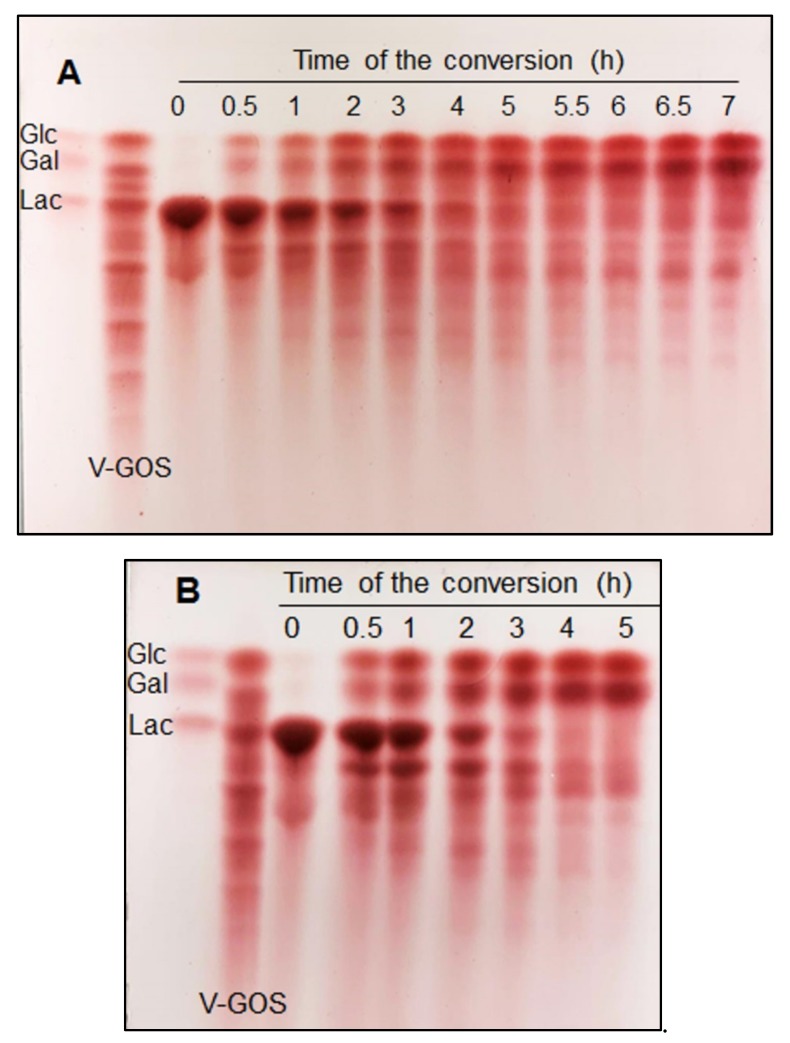
Hydrolysis of lactose catalyzed by recombinant β-galactosidases from *L. helveticus*, (**A**) crude enzyme *Lp609Lh*β-gal and (**B**) purified *EcoliBL21Lh*β-gal, as analyzed by thin layer chromatography (TLC) on pre-activated silica plates (eluent *n*-butanol/*n*-propanol/ethanol/water mixture: 2/3/3/2). The reactions were carried out at 50 °C with an initial lactose concentration of 205 g L^−1^ in 50 mM sodium phosphate buffer (pH 6.5) containing 1 mM MgCl_2_ and 1.5 U_Lac_ mL^−1^ of recombinant β-galactosidase. The samples were taken at regular time intervals during the reactions. A commercially available GOS preparation, Vivinal (Friesland Foods Domo), was added for comparison (indicated by V-GOS). A standard mixture of lactose (Lac), glucose (Glc) and galactose (Gal) was used as standards.

Both crude enzyme *Lp609Lh*β-gal and purified enzyme *EcoliBL21Lh*β-gal are more stable in the presence of high lactose concentration, retaining more than 75% and 60% of initial activity, respectively, after 24 h of incubation at 50 °C (data not shown). Thermostabilty of both crude enzyme and *Lp609Lh*β-gal and purified enzyme *EcoliBL21Lh*β-gal shows the potential of applications of these recombinant β-galactosidases in lactose conversion processes at temperatures up to 50 °C, whereas almost all lactobacillal LacLM-type β-galactosidases can be used in the applications, where the process temperatures can only be as high as 37 °C [7,8,9,10,11]. Figure 5 shows the formation of GOS by recombinant β-galactosidases from *L. helveticus*, crude enzyme *Lp609Lh*β-gal (Figure 5A) and purified enzyme *EcoliBL21Lh*β-gal (Figure 5B), as analyzed by thin layer chromatography (TLC) in discontinuous lactose conversion processes at 50 °C using 205 g L^−1^ initial lactose in 50 mM sodium phosphate buffer (pH 6.5) containing 1 mM MgCl_2_ and 1.5 U_Lac_ mL^−1^ of recombinant β-galactosidase. A commercial GOS preparation, Vivinal (Friesland Foods Domo), was used for comparison (indicated by V-GOS). The conversion was slower when using crude enzyme *Lp609Lh*β-gal than the conversion using purified enzyme *EcoliBL21Lh*β-gal as complete hydrolysis was obtained after 7 h and 4 h, respectively (Figure 5A,B). Recombinant β-galactosidases from *L. helveticus* was found to be a promising candidate for the production of GOS as a spectrum of GOS was formed when compared with V-GOS on TLC. It also can be seen clearly from TLC analysis that the highest GOS yields are obtained after 2 h of conversion using purified enzyme *EcoliBL21Lh*β-gal or 4 h when using crude enzyme *Lp609Lh*β-gal. As the reactions progress further, GOS are also subjected to hydrolysis when the substrate lactose becomes depleted (Figure 5A,B). Detailed analysis of the GOS mixture and the identification of the individual GOS formed in lactose conversion process using recombinant β-galactosidases from *L. helveticus* are ongoing in our laboratory. 

## 3. Materials and Methods

### 3.1. Bacterial Strains and Culture Conditions

The bacterial strains and plasmids used in this study are listed in Table 4. *L. helveticus* DSM 20075 (ATCC 15009) was obtained from the German Collection of Microorganisms and Cell Cultures (DSMZ; Braunschweig, Germany). *Lactobacillus plantarum* WCFS1, isolated from human saliva as described by Kleerebezem et al. [39], was originally obtained from NIZO Food Research (Ede, The Netherlands) and maintained in the culture collection of the Norwegian University of Life Sciences, Ås, Norway. *Lactobacillus* strains were cultivated MRS medium (Roth, Karlsruhe, Germany) at 37 °C without agitation. *Escherichia coli* NEB5α (New England Biolabs, Ipswich, MA, USA) and *E. coli* MB2159 (D-alanine auxotrophe) [40] were used as cloning hosts in the transformation of DNA fragments; whereas *E. coli* BL21 Star DE3 (Invitrogen, Carlsbad, CA, USA) and *E. coli* T7 Express (Novagen, Darmstadt, Germany) were used as the expression hosts. *E. coli* strains were grown in Luria-Bertani (LB) medium at 37 °C with shaking at 200 rpm. The antibiotic concentrations were 100 μg/mL ampicillin for *E. coli*, 10 μg/mL of chloramphenicol (Cm) for both *Lactobacillus* and *E. coli*, or 5 μg/mL and 200 μg/mL of erythromycin for *Lactobacillus* and *E. coli*, respectively. *E. coli* MB2159 and *L. plantarum* TLG02 were cultivated in the respective media supplemented with D-alanine (200 μg/mL). 

### 3.2. Chemicals and Enzymes

All chemicals and enzymes were purchased from Sigma (St. Louis, MO, USA) unless stated otherwise and were of the highest quality available. All restriction enzymes, Phusion high-fidelity DNA polymerase, T4 DNA ligase, and corresponding buffers were from New England Biolabs (Frankfurt am Main, Germany). Staining dyes, DNA and protein standard ladders were from Bio-Rad (Hercules, CA, USA). Isopropyl-β-d-thiogalactopyranoside (IPTG) was from Roth (Karlsruhe, Germany); whereas phenylmethanesulfonyl fluoride (PMSF) was obtained from Fluka (Buchs, Switzerland). Glucose (HK) Assay Kit for the determination of D-glucose was purchased from Megazyme (Bray, Ireland). 

### 3.3. DNA Manipulation

Genomic DNA of *L. helveticus* DSM 20075 was isolated using GenElute Bacterial Genomic DNA Kit from Sigma (St. Louis, MO, USA) according to the manufacturer’s instructions. The *lacLM* genes were amplified using Phusion high-fidelity DNA polymerase with the primer pairs FwdNcoI and Rev1XhoI, or FwdNcoI and Rev2XhoI (Table 5), which were designed based on the sequence of β-galactosidase from *L. helveticus* DSM 20075 with a GenBank accession number AJ512877. The primers were supplied by VBC-Biotech Service (Vienna, Austria) and the appropriate endonuclease restriction sites were introduced in the forward and reverse primers as indicated. The amplified PCR products were purified by the Monarch DNA Gel Extraction Kit (New England Biolabs). When needed, the PCR fragments were subcloned into the pJET1.2 plasmid (CloneJET PCRcloning kit, Fermentas), and *E. coli* NEB5α was used as a host for obtaining the plasmids in sufficient amounts. The sequence of the insert was confirmed by DNA sequencing performed by a commercial provider (Microsynth, Vienna, Austria).

### 3.4. Construction of Plasmids and Expression in E. coli

The fragment of *lacLM* genes, which was amplified with the primer pair FwdNcoI and Rev1XhoI (Table 5), was cloned into the expression vector pET-21d(+) (Novagen, Darmstadt, Germany) using the *Nco*I and *Xho*I cloning sites, resulting in the plasmid pET21lacLMLh. The constructed plasmid was transformed into electrocompetent cells of two different hosts, *E. coli* BL21 Star (DE3) and *E. coli* T7 Express harboring the plasmid pGRO7, which encodes the chaperones GroEL and GroES (Takara, Shiga, Japan), to compare the expression levels. 

The expression levels of β-gal without and with co-expression of the chaperones GroEL and GroES were compared. To this end, *E. coli* BL21 Star (DE3) harboring the plasmid pET21lacLMLh was grown in 100 mL of LB broth containing 100 μg/mL ampicillin at 37 °C to an OD_600_ of 0.6. Isopropyl-β-d-thiogalactopyranoside (IPTG) was then added to the culture medium and the cultures were incubated further at 25 °C for 12 h. Different induction conditions were compared by varying the concentrations of isopropyl-β-d-thiogalactopyranoside (IPTG) (0.1, 0.5 and 1 mM) in LB medium. *E. coli* T7 express GRO cells carrying the plasmid pET21lacLMLh was grown as described previously [25]. *E. coli* T7 express GRO cells carrying the plasmid pET21lacLMLh were grown at 37 °C in 100 mL LB medium containing 100 µg mL^−1^ ampicillin, 20 µg mL^−1^ chloramphenicol and 1 mg mL^−1^ L-arabinose until OD_600nm_ of 0.8 was reached. IPTG (1 mM) was then added into the culture, and the culture was incubated further at 18 °C. The cultures were harvested by centrifugation (4000 *g*, 4 °C for 15 min), washed twice and resuspended in 0.5 mL of 50 mM sodium phosphate buffer (NaPB), pH 6.5. Cells were disrupted on ice by sonication (Bandelin Sonopuls HD60, Berlin, Germany). The crude recombinant enzymes from *E. coli* BL21 Star (DE3) (*EcoliBL21Lh*β-gal) and *E. coli* T7 Express GRO (*EcoliGROLh*β-gal) were obtained after centrifugation (10,000× *g* for 15 min at 4 °C) and tested for β-galactosidase activity using the standard enzyme assays and protein concentrations.

### 3.5. Construction of Plasmids and Expression in L. plantarum

**Figure 6:**
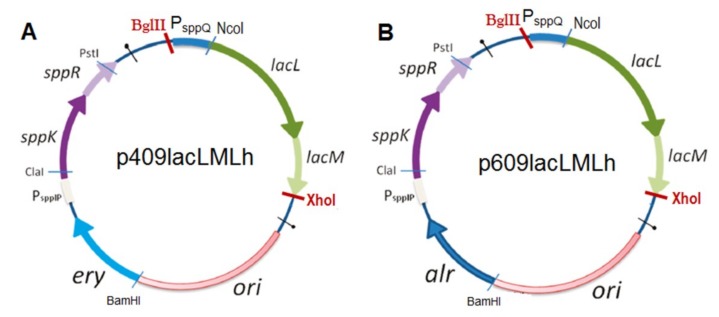
Expression vectors for *lacLM* from *L. helveticus* DSM 20075 in *L. plantarum* based on the erythromycin resistance gene (*erm*) [31] (**A**) and the alanine racemase gene (*alr*) [32] (**B**) as selection markers. *sppK* and *sppR*, denoting a histidine kinase and a response regulator, respectively, are regulated by P_sppIP_. Overlapping genes *lacLM* from *L. helveticus* are controlled by the inducible promoter PsppQ (pSIP409 derivatives).

The fragment of *lacLM* genes, which was amplified with the primer pair FwdNcoI and Rev2XhoI (Table 5), was cloned into the lactobacillal expression vector pSIP409 using the *Nco*I and *Xho*I cloning sites, resulting in the plasmid p409lacLMLh. The ~3.0 kb of *Bgl*II-*Xho*I fragment of p409LacLMLh containing the fragment of *lacLM* genes was ligated into the ~5.8 *Bgl*II-*Xho*I digested fragment of pSIP609 yielding the plasmid p609lacLMLh (Figure 6). The plasmids p409lacLMLh and p609lacLMLh were constructed in *E. coli* NEB5α and MB2159, respectively, before being transformed into the expression hosts, *L. plantarum* WCFS1 and the D-alanine auxotroph *L. plantarum* TLG02, respectively. The overexpression of β-gal was performed as described previously [12]. Overnight cultures of *L. plantarum* carrying the plasmids p409LacLMLh and p609LacLMLh, respectively, were inoculated into 500 mL of MRS broth (for *erm*-based vector p409LacLMLh 5 μg/mL erythromycin was added) to an OD_600_ of ~0.1. The cultures were incubated at 30 °C to an OD_600_ of 0.3 prior to induction with an inducing peptide pheromone IP-673 to a final concentration of 25 ng/mL. Cells were harvested at OD_600nm_ of 1.8 to 2.0. The cells were harvested by centrifugation (4000 *g*, 4 °C for 15 min), washed twice and resuspended in 0.5 mL of 50 mM sodium phosphate buffer (NaPB), pH 6.5. Cells were disrupted on ice by sonication (Bandelin Sonopuls HD60, Berlin, Germany). The crude recombinant enzymes from *L. plantarum* WCFS1, *Lp409Lh*β-gal, and *L. plantarum* TLG02, *Lp609Lh*β-gal, were obtained after centrifugation (10,000× *g* for 15 min at 4 °C) and tested for β-galactosidase activity using the standard enzyme assays and protein concentrations.

### 3.6. Fermentation and Purification of Recombinant β-Galactosidase

To obtain sufficient amount of enzyme for further characterization, *E. coli* BL21 Star (DE3) carrying the plasmid pET21lacLMLh was cultivated in 300 mL LB medium containing 100 µg mL^−1^ ampicillin at 37 °C in HT-Multifors fermentor (Infors HT, Bottmingen, Switzerland). The cultivation conditions and the induction protocol were identical to those described above for small-scale cultivations. Expression of *lacLM* was induced at OD_600_ of 0.6, the cells were harvested at OD_600_ ~6 and washed twice with 50 mM NaPB (pH 6.5), resuspended in the same buffer containing 1 mM PMSF and disrupted using a French press (Aminco, Silver Spring, MD, USA). Cell debris was removed by centrifugation (250,000× *g* for 30 min at 4 °C).

*EcoliBL21Lh*β-gal was purified using a prepacked 1 mL HisTrap HP Ni-immobilized metal ion affinity chromatography (IMAC) column (GE Healthcare, Uppsala, Sweden). Crude enzyme was loaded on the column that was pre-equilibrated with buffer A (20 mM NaPB, 20 mM imidazole, 500 mM NaCl, pH 6.5). The His-tagged protein was eluted at a rate of 1 mL min^−1^ with a 15 mL linear gradient from 0 to 100% buffer B (20 mM NaPB, 500 mM imidazole, 500 mM NaCl, pH 6.5). Active fractions were collected, desalted and concentrated by ultrafiltration using an Amicon Ultra Centrifugal Filter Unit with a 30 kDa cut-off membrane (Millipore, MA, USA). The purified *EcoliBL21Lh*β-gal was stored in 50 mM NaPB (pH 6.5) at 4 °C for further characterization. 

### 3.7. Gel electrophoresis Analysis

The purity and the molecular masses of recombinant β-galactosidase *EcoliBL21Lh*β-gal was determined by SDS-PAGE, in which the enzyme (~1 mg_protein_ mL^−1^) was incubated with Laemmli buffer at 90 °C for 5 min. Protein bands were visualized by staining with Bio-safe Coomassie (Bio-Rad). The determination of protein mass was carried out using Unstained Precision plus Protein Standard (Bio-Rad).

### 3.8. β-Galactosidase Assays

β-Galactosidase activity was determined using *o*-nitrophenyl-β-d-galactopyranoside (*o*NPG) or lactose as the substrates as previously described [7]. When chromogenic substrate *o*NPG was used, the reaction was started by adding 20 μL of enzyme solution to 480 μL of 22 mM *o*NPG in 50 mM NaBP (pH 6.5) and stopped by adding 750 μL of 0.4 M Na_2_CO_3_ after 10 min of incubation at 30 °C. The release of *o*-nitrophenol (*o*NP) was measured by determining the absorbance at 420 nm. One unit of *o*NPG activity referred to the amount of β-galactosidase liberating 1 μmol of *o*NP per minute under the defined conditions. 

When the natural substrate lactose was used, 20 μL of enzyme solution was added to 480 μL of 600 mM lactose solution in 50 mM NaBP (pH 6.5). The reaction was stopped after 10 min of incubation at 30 °C by heating the reaction mixture at 99 °C for 5 min. After being cooled to room temperature, the release of D-glucose in the reaction mixture was determined using Glucose (HK) Assay Kit (Megazyme) according to the manufacturer’s instructions. One unit of lactase activity referred to the amount of β-galactosidase liberating 1 μmol of D-glucose per minute under the defined condition. 

### 3.9. Determination of Protein Concentration

Protein concentrations were determined using the method of Bradford [41] with bovine serum albumin (BSA) as a standard.

### 3.10. Biochemical Characterization of Recombinant β-Galactosidases

All steady-state kinetic measurements using *o*NPG and lactose as the substrates were conducted in 50 mM NaPB (pH 6.5) at 30 °C, with concentrations ranging from 0.5 to 22 mM for *o*NPG and from 1 to 600 mM for lactose, respectively. The kinetic parameters were calculated by nonlinear regression, in which the observed data were fit to the Henri-Michaelis-Menten equation using data analysis software (SigmaPlot, SPSS, Chicago, IL, USA).

The dependence of enzyme activity on pH and temperature was evaluated by performing the enzyme assays using *o*NPG and lactose as the substrates under the specified conditions. The pH dependency test was conducted in Britton-Robinson buffer (mixture of 20 mM each of acetic, phosphoric and boric acids adjusted with 1 M NaOH to the desired pH) with the pH range of 4–9 at 30 °C, while the temperature dependency test was conducted in 50 mM NaPB (pH 6.5) at the temperature range of 20–80 °C. The pH stability was determined by incubating the enzyme at various pH values (pH 4–9) using Britton-Robinson buffer at 30 °C. At certain time intervals, samples were withdrawn. The residual *o*NPG activity was measured, and the half-life (*τ*_1/2_) value of pH inactivation was obtained when the residual *o*NPG activity decreases to 50% of its initial value. The thermostability was determined by incubating the recombinant enzyme in 50 mM NaPB (pH 6.5) at various temperatures ranging from 4–80 °C. The effect of magnesium ion (Mg^2+^) on thermostability of the enzyme was investigated by incubating the enzyme in the presence of 1 mM MgCl_2_ at 40 and 50 °C. The residual *o*NPG activity was measured at certain time intervals, and *τ*_1/2_ value was determined.

To study the effect of various cations on the release of *o*NP from *o*NPG, the enzyme samples were assayed with 22 mM *o*NPG in 10 mM Bis-Tris (pH 6.5) in the presence of various cations with final concentrations of 1.0, 10, and 100 mM (chloride or sulfate form), at 30 °C for 10 min. The measured activities were compared with the activity of the enzyme solution without added cations under the same conditions.

### 3.11. Formation of Galacto-Oligosaccharides (GOS)

The formation of GOS was carried out in discontinuous mode using recombinant β-galactosidases from *L. helveticus*, crude enzyme *Lp609Lh*β-gal and purified enzyme *EcoliBL21Lh*β-gal. Reaction conditions were 205 g L^−1^ initial lactose concentration in 50 mM phosphate buffer (pH 6.5) at 50 °C, 1.5 U_Lac_ per mL of reaction mixture and constant agitation (300 rpm). Samples were withdrawn at intervals and the GOS mixtures formed were analyzed by thin layer chromatography (TLC). TLC was carried out using high-performance TLC silica plates (Kieselgel 60 F245, Merck). An appropriately diluted sample containing ~20 g L^−1^ sugar was applied to the plate (1.0 µL) and eluted twice in ascending mode with an *n*-butanol/*n*-propanol/ethanol/water mixture (2/3/3/2). Thymol reagent was used for detection.

### 3.12. Statistical Analysis

All experiments and measurements were conducted at least in duplicate, and the percentage error was always less than 5%. 

## 4. Conclusions

β-Galactosidase encoding genes *lacLM* from an industrially important organism *L. helveticus* DSM 20075 were cloned and successfully overexpressed in *E. coli* and *L. plantarum* using different expression systems. Although the co-expression of the chaperones GroEL/GroES did not show an increase in expression levels, the expression of this LacLM-type β-galactosidase was greatly enhanced through recombinant production in *E. coli*. The expression in the *L. plantarum* using food-grade expression vector shows the potential for the production of food-grade enzyme for applications in the dairy industry. The detailed information on the properties of recombinant β-galactosidase from *L. helveticus* provided in the present study can serve as the basis for the applications of this biotechnologically attractive enzyme. This enzyme exhibited high transgalactosylation activity and revealed interesting properties for applications in lactose conversion and GOS formation processes. Currently, we are working on the synthesis of GOS using β-galactosidase from *L. helveticus* and the identification of the GOS formed.

## Figures and Tables

**Table 1:** Kinetic parameters of recombinant β-galactosidase from *L. helveticus* (*EcoliBL21Lh*β-gal) for the hydrolysis of *o*-nitrophenyl-β-d-galactopyranoside (*o*NPG) and lactose.

Substrate	Method for Determination of β-Galactosidase Activity	Kinetic Parameter	*EcoliBL21Lh*β-Gal
*o*NPG	Release of *o*NP	*v*_max,*o*NP_ (μmol min^−1^ mg^−1^)	476 ± 66
		*K*_m,*o*NPG_ (mM)	1.40 ± 0.30
		*k*_cat_ (s^−1^)	865 ± 120
		*k*_cat_/*K*_m_ (mM^−1^ s^−1^)	618
		*K*_i,s_ (mM)	3.6 ± 0.8
Lactose	Release of D-glucose	*v*_max,Glucose_ (μmol min^−1^ mg^−1^)	11.1 ± 0.2
		*K*_m,Lactose_ (mM)	15.7 ± 1.3
		*k*_cat_ (s^−1^)	20.2 ± 0.3
		*k*_cat_/*K*_m_ (mM^−1^ s^−1^)	1.29

**Table 2:** Effect of pH (**A**) *^a^* and temperature (**B**) *^b^* on catalytic stability of recombinant β-galactosidase from *L. helveticus* expressed in *E. coli* (*EcoliBL21Lh*β-gal).

(A)	pH	*τ*_1/2_*^c^* (h)	(B)	Temperature (°C)	*τ*_1/2_ (h)
	4.0	1		4	6 months
	5.0	8		20	2 months
	5.5	816		30	216
	6.0	552		40	5
	6.5	144		50	0.33
	7.0	48		40 (+1 mM MgCl_2_)	72
	8.0	1		50 (+1 mM MgCl_2_)	3
	9.0	0.5			

*^a^* The pH stability was determined by incubating the enzyme at various pH values (pH 4–9) using Britton-Robinson buffer at 30 °C. *^b^* Temperature stability was determined by incubating the recombinant enzyme in 50 mM NaPB (pH 6.5) at various temperatures ranging from 4–80 °C. *^c^* The values for half-life time of activity (*τ*_1/2_) were calculated as the mean of two independent experiments, and the percentage error was always less than 5%.

**Table 3:** Effect of various cations on activity of recombinant β-galactosidase from *L. helveticus* expressed in *E. coli* (*EcoliBL21Lh*β-gal).

Cation	Relative Activity (%)		
	1 mM	10 mM	100 mM
None	100	100	100
Na^+^	160	142	109
K^+^	118	96	68
K^+^ *^a,c^*	204	204	nd
Mn^2+^ *^a,c^*	143	105	nd
Mg^2+^ *^a,c^*	152	117	nd
Ca^2+^ *^a,c^*	140	80	nd
Cu^2+^ *^b,c^*	17	7	nd
Zn^2+^ *^a,c^*	120	78	nd

*^a^* added as chloride salt; *^b^* added as sulfate salt; *^c^* the reaction was performed in the presence of 1 mM Na^+^; nd, not determined.

**Table 4:** Strains and plasmids used for cloning and overexpression of β-galactosidase encoding genes *lacLM* from *L. helveticus ^a^*.

Strain or Plasmid	Relevant Characteristics	Reference
**Strains**		
*L. helveticus* DSM 20075	source of *lacLM* genes	DSMZ (Braunschweig, Germany)
*L. plantarum* WCFS1	wild-type, expression host	[39]
*L. plantarum* TLG02	Δ*alr*, D-alanine auxotroph, expression host	[32]
*E. coli* NEB5α	cloning host	New England Biolabs (Ipswich, MA)
*E. coli* MB2159	D-alanine auxotroph, cloning host	[40]
*E. coli* BL21 Star DE3	expression host	Invitrogen (Carlsbad, CA)
*E. coli* T7 Express	expression host	Novagen (Darmstadt, Germany)
**Plasmids**		
pET-21d(+)	T7 Promoter, Amp^r^, C-terminal His-Tag	Novagen (Darmstadt, Germany)
pGRO7	plasmid encoding chaperones GroES and GroEL, Cm^r^	Takara (Shiga, Japan)
pSIP409	*spp*-based expression vector, Erm^r^, *gusA* controlled by P_sppQ_	[30]
pSIP609	derivative of pSIP409, *erm* replaced by *alr*	[32]
pET21lacLMLh	*lacLM* from *L. helveticus* in pET-21d(+)	current study
p409lacLMLh	pSIP409 derivative, containing *lacLM* from *L. helveticus*	current study
P609lacLMLh	pSIP609 derivative, containing *lacLM* from *L. helveticus*	current study

*^a^ lacLM*, β-galactosidase genes; Δ*alr*, D-alanine auxotroph; Amp^r^, ampicillin resistance; Erm^r^, erythromycin resistance; Cm^r^, chloramphenicol resistance; *alr*, alanine racemase encoding gene; *erm*, erythromycin resistance gene; *spp*, sakacin P gene cluster; *gusA*, β-glucuronidase reporter gene.

**Table 5:** Oligonucleotide primers used for amplification of β-galactosidase encoding genes *lacLM* from *L. helveticus ^a^*.

Primer	Sequence (5’-3’)	Reference Accession Number
FwdNcoI	GGCCCCATGGAAGCAAATATCAATTG	GenBank: AJ512877
Rev1XhoI	GGCCCTCGAGTGAATTTAGAATAAATGAAAATTC	GenBank: AJ512877
Rev2XhoI	GGCCCTCGAGTTAATTTAGAATAAATGAAAATTC	GenBank: AJ512877

*^a^* Restriction sites are underlined.
